# Locating Creativity in Differing Approaches to Musical Robotics

**DOI:** 10.3389/frobt.2021.647028

**Published:** 2021-03-23

**Authors:** Steven Kemper

**Affiliations:** Music Department, Mason Gross School of the Arts, Rutgers University, New Brunswick, NJ, United States

**Keywords:** robotic musical instruments, creativity, anthropomorphism, music generation, musical robotics

## Abstract

The field of musical robotics presents an interesting case study of the intersection between creativity and robotics. While the potential for machines to express creativity represents an important issue in the field of robotics and AI, this subject is especially relevant in the case of machines that replicate human activities that are traditionally associated with creativity, such as music making. There are several different approaches that fall under the broad category of musical robotics, and creativity is expressed differently based on the design and goals of each approach. By exploring elements of anthropomorphic form, capacity for sonic nuance, control, and musical output, this article evaluates the locus of creativity in six of the most prominent approaches to musical robots, including: 1) nonspecialized anthropomorphic robots that can play musical instruments, 2) specialized anthropomorphic robots that model the physical actions of human musicians, 3) semi-anthropomorphic robotic musicians, 4) non-anthropomorphic robotic instruments, 5) cooperative musical robots, and 6) individual actuators used for their own sound production capabilities.

## Introduction

The field of musical robotics presents an interesting case study of the intersection between creativity and robotics. While the potential for machines to express creativity represents an important issue in the field of robotics and AI, this subject is especially relevant in the case of machines that replicate human activities that are traditionally associated with creativity, such as music making. Several recent studies have explored the history and current state of musical robotics. While these present an overview of the field, they tend to focus primarily on issues related to functional design, with little discussion of creativity. Musical robots are categorized based on how they produce sound (Kapur 2005), how they function as interactive multimodal systems (Solis and Ng 2011), how they developed over history (Murphy et al., 2012; Long et al., 2017), and the ways that they engage in “Robotic Musicianship” (Bretan and Weinberg, 2016).^1^

Based on a review of existing literature as well as the author’s experience designing and composing music for musical robots, this article proposes a new classification framework based on the ways that musical robots express creativity through anthropomorphic form, capacity for sonic nuance, control, and musical output. By exploring the field of musical robotics through this lens, we are able to better understand the ways that specific approaches lead to both technical and artistic goals.

## Definitions and Criteria for Evaluation

### Defining Musical Robotics

Both designers and audiences use the term “musical robots” or “robotic musical instruments” to refer to a broad range of musical machines. From an engineering perspective, approaches that lack autonomy could be more accurately be described as “musical mechatronics” (Bretan and Weinberg, 2016). However, the popular conception of robots, rooted in mythology, includes any machines that can mimic human actions (Jones 2017; Szollosy, 2017). Therefore, this discussion will consider “musical robotics” as any approach where an electromechanical actuator produces a visible, physical action that models the human act of music making, regardless of autonomous control.

By modeling the human act of music making, musical robots may be considered inherently anthropomorphic. Fink describes the important connection between anthropomorphism and robotics, as expressed through anthropomorphic form (appearance), behavior, and interaction with humans (Fink, 2012). While not all musical robots possess an anthropomorphic form, modeling the physical actions of music making represents anthropomorphic behavior. The ways that designers and audiences experience anthropomorphism significantly impacts how these machines express creativity. With this idea in mind, I identify six approaches that express creativity in different ways. These include: 1) nonspecialized anthropomorphic robots that can play musical instruments, 2) specialized anthropomorphic robots that model the physical actions of human musicians, 3) semi-anthropomorphic robotic musicians, 4) non-anthropomorphic robotic instruments, 5) cooperative musical robots, and 6) individual actuators used for their own sound production capabilities.

### Defining Creativity

Several different fields currently focus on creativity, including esthetics, psychology, and artificial intelligence (Götz 1981; Bailin 1983; Boden, 1996; Boden, 2004; Cope 2005; Runco and Jaeger, 2012). Runco and Jaeger distinguish two fundamental criteria of creativity: originality and effectiveness (Runco and Jaeger 2012, 92). Originality, or creative insight, emerges from what Cope describes as, “The initialization of connections between two or more multifaceted things, ideas, or phenomenon hitherto not otherwise considered actively connected” (Cope 2005, 11). Effectiveness is determined through evaluation of creative insight by the creator, as well as related communities (Boden, 1996, 268).

### Evaluative Criteria for Creativity in Musical Robotics

Musical robots tend to be viewed as creative machines due to their connection to music, which is understood to be an inherently creative endeavor. While studies of creativity in musical robots should focus on the music they produce, originality and effectiveness are also expressed through anthropomorphic form, capacity for sonic nuance, control, as well as musical output.

#### Anthropomorphic Form

The physical appearance of musical robots as well as the ways they model the human actions of music making are extremely important for designers and audiences. According to Fink, “the physical shape of a robot strongly influences how people perceive it and interact with it…” (Fink, 203). Fink also describes the importance of anthropomorphic behavior from the observer’s perspective. “If a system behaves much like a human being (e.g., emits a human voice), people’s mental model of the system’s behavior may approach their mental model of humans,” based on the estimation of the robot’s capabilities (Fink, 201). Some approaches to musical robotics focus on modeling human appearance and movement while others explore mechatronic sound production techniques that do not possess anthropomorphic form. Evaluating creativity in terms of anthropomorphic form requires an understanding of the ways that designers and audiences ascribe human qualities to a musical robot’s form and behavior.

#### Capacity for Sonic Nuance

Much of the existing literature in the field of musical robotics focuses on robots’ ability to model the sonic capabilities of human performers. The benchmark for success in this area is often described as the ability to play music expressively (e.g., Murphy, 2014). While designers often describe how advancements in sound control parameters and their resolution enable expressivity, the concept of expression tends to be loosely defined (Kemper and Cypess, 2019). Therefore, it is more accurate to describe these features as increasing the capacity for sonic nuance (Kemper and Barton, 2018). While greater capacity for sonic nuance allows musical robots to more accurately model the dynamics, articulations, and phrasing of human performers, it can also create novel sonic and musical possibilities that differ from the ways that humans perform (Kemper, 2014). Thus, creativity in this domain refers to novel approaches to sonic nuance either for the purposes of modeling human performance or exploring new sonic and musical possibilities that are unique to musical robots.

#### Control

Musical robots can be controlled in a variety of ways, ranging from autonomous modes that enable interaction with human performers to modes where the movement of every actuator is preprogrammed. One of the challenges of assessing creativity in musical robotics is that control systems are often separable from the robot itself. Research in the areas of artificial musical generation and listening algorithms tend to focus on note generation in a generic way (e.g., as MIDI data), rather than being tailored to the mechanical requirements of a specific robot (e.g., Cope 2005; Xia and Dannenberg, 2015). For example, Solis and Ng’s *Musical Robots and Interactive Multimodal Systems,* is divided into two separate sections that describe control and output respectively (Solis and Ng, 2011). While control determines the ways that actuators operate and thus how the robot produces sound, it is important to distinguish these instructions from the actual musical output.

#### Musical Output

The music that robots perform represents an important avenue for expressing creativity; however, this topic has received surprisingly little attention. Some robots use a single piece of music to demonstrate their capabilities, while others perform in a diverse array of styles, collaborate in real time with human performers, and are designed as creative tools for musical artists.^2^ Evaluating creativity in musical output should consider both the robot’s performative capabilities and musical decisions. Performative capabilities include the ability for robots to present a compelling performance, either by modeling human performers or exploring their own unique capabilities. Musical decisions include the specific musical pieces composed or arranged for the robot(s), musical decision-making by autonomous control systems, and the ways that new music created for (or by) robots engages with the unique capabilities of these machines.

## Different Approaches to Musical Robotics

### Nonspecialized Anthropomorphic Robots that Can Play Musical Instruments

Over the past 2 decades several companies have developed general-purpose anthropomorphic bipedal robots that replicate human actions in a variety of areas, including musical performance (Goswami and Vadakkepat 2019). For example, Toyota modified versions of their Partner robot to play trumpet, violin, and an electronic drum kit (Doi and Nakajima 2019). Of these approaches, the trumpet robot approximates human performance most closely in terms of articulation, dynamics, and timing. Conversely, the violin playing robot is limited in its range, and struggles somewhat with intonation and tone compared to a trained violinist.^3^ This reflects the challenges of modeling the complex physical actions of bow pressure, bow speed, proper finger position, and vibrato.

In general, these demonstrations prioritize showing versatile, humanoid robots engaging in a quintessentially “human” activity over novel musical output. As Doi and Nakajima state, “We began the development of musical performance humanoid out of curiosity that we would like to make a humanoid robot realize such a human unique activity [*sic*]” (Doi and Nakajima, 218). This is emphasized by the fact that available videos of these robots perform easily recognizable versions of popular music, including “When you Wish Upon a Star” and “Pomp and Circumstance.“^4^,^5^ As robots are more specifically designed for musical performance, they become more specialized in their ability to produce sonic nuance as described in the examples below.

### Specialized Anthropomorphic Robots That Model the Physical Actions of Human Musicians

Several approaches have focused on building robots that model the physical actions involved in musical performance. These include pioneering work from Waseda University, including the WABOT-series piano robot, WF-series flutist robot, and the WAS-series saxophone robot (Roads 1986; Solis et al., 2006; Solis and Hashimoto 2010). Shibuya and Park have also created robotic models of violin performance (Shibuya et al., 2007; Park et al., 2016), and Chadefaux has created a robotic “finger” for harp plucking (Chadefaux et al., 2012).

While these approaches accurately model the actions of human performance, they can result in a lack of musical “efficiency” when compared to musical robots that do not model human actions (see *Non-anthropomorphic Robotic Instruments*). For example, the Waseda WF-4RII Flutist Robot possesses 43 DOF, and each robotic component is designed to replicate its human counterpart, including “humanoid organs” such as robotic lips, lungs, arms, neck, tongue, and oral cavity (Solis et al., 2006, 13). By modeling the human actions of performance, the robot helps us to understand how instrumental performers produce musical sounds. However, the complexity of the mechanical model limits the sonic possibilities that are available to machines, such as super-virtuosic speed and novel approaches to sonic nuance. This is evidenced by available videos of performance, such as that of the WF-4RII performing Rimsky-Korsakov’s “Flight of the Bumblebee” at a (humanly) comfortable tempo of c.150 BPM.^6^

### Semi-Anthropomorphic Robotic Musicians

The musical robots in this category assume an anthropomorphic form, however they do not model the specific actions of human performance and are focused more on appearance and musical output. Over the past few decades several robotic “bands” have emerged, including the rock bands *The Trons, Captured! By Robots* and *Compressorhead*, as well as a collaboration between *Z-Machines* ^7^ and Squarepusher on the 2014 album “Music for Robots.” (Snake-Beings, 2017; Gallagher, 2017; Davies and Crosby, 2016; Squarepusher x Z-Machines, 2014). *MOJA* features a drummer, harpist, and flutist performing in a style evocative of traditional Chinese music.^8^ In addition to these “bands,” the Robotic Musicianship Group at Georgia Tech has developed two well-documented semi-Anthropomorphic musical robots: Haile, a robotic drummer, and Shimon, a robotic marimba player (Weinberg and Driscoll 2006; Weinberg et al., 2020).

The anthropomorphic nature of these robots is highlighted primarily by their stylized appearance, rather than an attempt to model the human actions of performance. For example, *Compressorhead*’s multi-armed drummer “Stickboy” has a mohawk made of metal spikes and is designed to headbang along with the music. *MOJA*’s robots are dressed in Tang-dynasty style garments. These design choices have nothing to do with sound production, however they enhance the connection between the audience and robot performers.

Even though the sound-producing mechanisms of these robots do not model human performers, much of the music they play could be easily performed by humans. One exception to this is Squarepusher’s approach*,* which engages with the unique musical possibilities afforded by *Z-Machines’s* robots. In the song “Sad Robot Goes Funny” the double-necked “guitar-bot” instrument performs extremely rapid picking while at the same time dynamically changing the chords in a way that would be impossible for a human musician. This takes full advantage of that instrument’s 78 solenoid-based “fingers” and picks that can articulate each string individually. Similarly, while Shimon and Haile are designed to perform with human musicians, both have explored the extra-human musical capabilities of their designs with an emphasis on “play [ing] like a machine” (Weinberg et al., 2020, 95).

### Non-Anthropomorphic Robotic Instruments

Non-anthropomorphic robotic instruments can either be mechatronic augmentations of existing acoustic instruments (e.g. Yamaha’s Disklavier),^9^ or newly designed instruments with no acoustic analog (e.g. Andy Cavatorta Studio’s Gravity Harp).^10^ Table 1 includes a selection of recently active groups and individuals producing collections of non-anthropomorphic robotic instruments, as well as well-documented individual robots.

**TABLE 1:** Selection of recently active groups and individuals producing collections of non-anthropomorphic robotic instruments, as well as recently designed, well-documented individual robots.

Group name	Website	Citation
Andy Cavatorta Studio	https://andycavatorta.com/	n/a
EMMI: Expressive Machines Musical Instruments	https://www.youtube.com/user/ExpressiveMachines	Rogers et al. (2015)
Karmetic Machine Orchestra	https://www.karmetik.com/karmetik-projects/the-machine-orchestra/	Kapur et al. (2011)
LEMUR: League of Electronic Musical Urban Robots	http://lemurbots.org/index.html	Singer et al. (2004)
Logos Foundation Man and Machine Orchestra	https://www.logosfoundation.org/mnm/index.html	Maes et al. (2011)
Matthew Steinke	https://www.matthewsteinke.com/robotic	n/a
Maywa Denki	https://www.maywadenki.com/sketch/tsukuba-2/	Ippolito (2009)
Moritz Simon Geist	http://sonicrobots.com/	n/a
Music, Perception and Robotics Lab	https://mprlab.org/	Barton et al. (2018)
Robot Rickshaw	http://www.troy82.com/musical-robots/projects/robot-rickshaw/	n/a
Sonic Engineering Lab for Creative Technology	https://ecs.wgtn.ac.nz/Groups/SELCT/IndexProto	Murphy (2014)
Studio Jose de la O	http://delao.mx/robokumbia	Flores et al. (2019)
Trimpin	https://www.historylink.org/File/11204	Leitman (2011)

Individual Robots	Website	Citation
Berimbot	http://www.berimbo.com.br/	Monsão et al. (2017)
Gamelatron	https://gamelatron.com/	McGraw (2016)
McBlare	https://www.cs.cmu.edu/∼music/mcblare/	Dannenberg et al. (2011)
MechDrum	https://mechdrum.wordpress.com/	Van Rooyen et al. (2017)

Non-anthropomorphic robotic instruments tend to focus more on sonic nuance than modeling the human actions of performance. For example, the Logos Foundation’s robotic vibraphone <Vibi> couples actuating and dampening solenoids to each bar of the instrument rather than designing robotic arms and hands with multiple degrees of freedom that would model a human performer (Maes et al., 2011, 41).^11^ This design allows <Vibi> to play much more rapidly than a human performer. It also enables complete polyphony of the instrument as well as individual control of the dampening mechanisms. <Vibi>‘s unique capabilities open up a new world of musical possibilities when compared to a human performer on a traditional instrument.

One drawback of <Vibi>‘s design is that since the solenoids are mounted below the striking bars it is difficult for the audience to see their movement, obscuring the connection between physical action and sound production. While this is a common issue in this category, some projects are designed to maximize the visibility of movement. For example, LEMUR’s GuitarBot features four vertically mounted strings where pitch is changed on each string with a belt-driven fret that travels over half a meter (Singer et al., 2003). Other approaches include using LEDs to visualize sound production (e.g. Rogers et al., 2015).

### Cooperative Musical Robots

An emerging area of musical robotics combines human performance and robotic actuation on a single shared interface. Barton describes these devices as cooperative musical machines, differentiating between cooperative (electro)mechanical instruments that do not react to human input, and cooperative robotic instruments that respond and interact with human performers (Barton, et al., 2017). Examples of cooperative (electro)mechanical instruments include Meywa Denki’s *Ultra Folk* acoustic guitar^12^ and Gurevich’s *STRINGTREES* (Gurevich, 2014). Examples of cooperative robotic instruments include Barton’s *Cyther*, a human-playable, self-tuning robotic zither, as well as the previously discussed Halie (Weinberg et al., 2020, 26).

Moving beyond a shared interface, Georgia Tech’s Robotic Drumming Prosthetic Arm robotically augments the capabilities of the body. This device consists of a prosthetic arm outfitted with brushless gimbal motors and a single stage timing belt drive connected to a drumstick (Weinberg et al., 2020, 219). An amputee drummer controls the stick through EMG sensors connected to muscles on the residual limb. Rather than simply serving as a replacement for a human arm, the capabilities of mechatronic design and robotic control, including the addition of a second stick, allow for humanly impossible virtuosity and speed (Weinberg et al., 213, 226). Beyond musical possibilities, robotic augmentation of the body concretizes notions of posthumanism and the cyborg (Haraway 1991). It also causes observers to question the “humanness” of an augmented individual rather than evoking a sense of anthropomorphism (Swartz and Watermeyer 2008), though that may change as these technologies become more widely accepted.

### Individual Actuators Used for Their Own Sound Production Capabilities

The final category in this discussion encompasses projects that focus on the sounds and movement of individual actuators. While some may not consider these approaches to be musical robots due to a lack of complexity in design or sonic output, I argue that they are important to consider in this discussion because 1) as with all of the other approaches described here, they possess electromechanical actuators that produce a visible, physical action resulting in sound production and 2) they distill sound produced by electromechanical actuators to its most basic form.

Several designers have created music using voice coil and stepper actuators from floppy disc and hard drives, as well as from individual stepper motors. These approaches tend to reproduce well-known music, such as Zadrożniak’s arrangement of the “Imperial March” from *Star Wars* for floppy disc drives.^13^ While these actuators produce a unique timbre, there is limited capacity for sonic nuance.

Other designers create work that produces sound using the simple actions of motors. For example, Zimoun builds large-scale sound sculptures that feature individual motors actuating resonant objects.^14^ One installation consists of 658 cardboard boxes that are hit by cotton balls connected to a DC motor by piano wire. As the motor spins the ball hits the box and produces a resonant sound. In this approach, gravity, friction, and resonance, as well as the movement of the motor itself produce variations in the sound that makes the work compelling.

## Discussion

This paper proposes a novel classification system that enables us to consider how different approaches to musical robotics express creativity in different ways. In general, the more overtly anthropomorphic the form, the more central the physical appearance of the project is to its creativity. For example, the Toyota Partner robot’s creative impact stems from the fact that a humanoid robot is performing a quintessentially human activity. As anthropomorphic form diminishes, originality and effectiveness are conveyed through the capacity for sonic nuance, as well as the ways that these machines either accurately model human performance or develop their own robotic performance practice. For an extreme case such as Zimoun’s work, the connection to anthropomorphic behavior in the process of sound production lies at the center of the work. By understanding the connections and divergent goals among different approaches to musical robotics, we can better evaluate the ways that these machines express originality and effectiveness for both designers and audiences. Though the classifications developed here are theoretical in nature, they will hopefully prove useful in developing future studies that explore the ways that musical machines can be considered creative.

## Data Availability

The original contributions presented in the study are included in the article/Supplementary Material, further inquiries can be directed to the corresponding author.

## Footnotes

1 This article defines Robotic Musicianship as the intersection of musical mechatronics and machine musicianship. While the concepts of creativity and anthropomorphism are mentioned in passing, they are not used to classify different approaches to musical robotics.

2 E.g. https://www.patmetheny.com/orchestrioninfo/

3 Toyota Partner Violin Robot: https://www.youtube.com/watch?v=-yInphJdick

4 Toyota Partner Trumpet Robot: https://www.youtube.com/watch?v=6fctULDctuA

5 Toyota Partner Violin Robot (see n.3).

6
http://www.takanishi.mech.waseda.ac.jp/top/research/music/flute/wf_4rii/index.htm (Section IV)

7
https://www.yurisuzuki.com/design-studio/z-machines

8
https://news.tsinghua.edu.cn/en/info/1012/5231.htm

9
https://usa.yamaha.com/products/musical_instruments/pianos/disklavier/index.html

10
https://andycavatorta.com/gravityharps.html

11
https://logosfoundation.org/instrum_gwr/vibi.html

12
https://www.maywadenki.com/sketch/tsukuba-2/

13
https://www.youtube.com/watch?v=yHJOz_y9rZE

14
https://www.zimoun.net/
